# Ecological stoichiometry, salt ions and homeostasis characteristics of different types of halophytes and soils

**DOI:** 10.3389/fpls.2022.990246

**Published:** 2022-10-13

**Authors:** Yinghan Zhao, Tian Li, Junhan Liu, Jingkuan Sun, Ping Zhang

**Affiliations:** ^1^ Shandong Key Laboratory of Eco-Environmental Science for Yellow River Delta, Binzhou University, Binzhou, China; ^2^ College of Forestry, Shandong Agricultural University, Taian, China; ^3^ College of Life Sciences, Ludong University, Yantai, China

**Keywords:** ecological chemometrics, halophyte, salt ions, homeostasis, Yellow River delta

## Abstract

Studying eco-stoichiometric and salt ions characteristics of halophytes and soils is helpful to understand the distribution mechanism of nutrients and salts in halophytes and their adaptation strategies to salinized habitats. In this study, three different types of halophytes (*Phragmites communis*-salt repellent, *Suaeda salsa*-salt accumulating, and *Aeluropus sinensis*- salt secreting) and soils were selected to analyze the differences and correlations of C, N, P stoichiometry and salt accumulation. Results showed that: (1) the total nitrogen (TN) and total phosphorus (TP) contents of the three halophytes’ leaves were significantly higher than those of the roots and stems, and the C: N ratios were contrary to the difference mentioned above. The growth of *P. communis* and *S. salsa* was mainly limited by P, whereas *A. sinensis* was limited by both N and P. *S. salsa* had a stronger absorption capacity for Na^+^ and Mg^2+^ than *P. communis* and *A. sinensis.* The interrelationship between salt ions and C, N and P ecological stoichiometry of halophyte organs was influenced by the type of halophytes. (2) The TC, TN, and N: P contents of the three halophyte communities in the surface soil (0-20 cm) were significantly higher than the other soil layers, while P did not differ significantly among soil layers. The planting of different halophytes affected the TC, TN, C: N, N: P values and the content of seven ions in the surface soil. SO_4_^2-^was positively correlated with soil TC, TN, N:P, and Na^+^ were positively correlated with soil TC in three halophytes. (3) The *P. communis* TC and *A. sinensis* TN contents were negatively correlated with soil TC, TN, C: P, and N: P, whereas TC contents of *S. salsa* were positively correlated with the aforementioned soil indicators. The *P. communis* and *A. sinensis* TC contents were negatively correlated with soil K^+^, while this correlation was opposite between *S. salsa* and soil. (4) The homeostasis of C, N, and P elements in all three halophytes showed that C > N > P, the homeostasis was strongest in *A. sinensis* and weakest in *S. salsa*. The results provide a theoretical basis for the restoration of saline land in the Yellow River Delta.

## 1 Introduction

In terrestrial ecosystems, the balance of energy and nutrients is called ecological stoichiometry (Yang et al., 2018), which represents the demand of organisms for natural resources and mainly connects biogeochemical cycles at different levels through carbon(C), nitrogen(N), and phosphorus(P) (Hu et al., 2018). Stoichiometric homeostasis is the core of ecological chemometrics (Koojiman, 1995), which refers to the ability of organisms to maintain their chemical composition relatively constant by adjusting the concentration of chemical elements and the proportion of different chemical elements in their organisms when external environmental conditions change. The strength of homeostasis can reflect the adaptability of organisms to environmental changes (Sterner and Elser, 2002). The elements C, N, and P are the basis for the composition of all living substances on earth (Jordi et al., 2012). For plants, C constitutes the basic structure of plants, accounting for about 50% of their biomass (Schade et al., 2003; Liu et al., 2011); N is the basic component of enzymes and plays a crucial role in plant production and photosynthesis (Elser et al., 2000); P is an essential element for nucleic acids and cell membranes, responsible for the composition of cellular structural DNA and RNA (Tilman, 2004; Bai et al., 2012).

Plant and soil ecological stoichiometric ratios are used to represent important ecological processes in terrestrial ecosystems. For example, the concept of leaf N: P ratios to assess nutrient limitations in plant growth has been demonstrated in a variety of plants (Koerselman and Meuleman, 1996; Schreeg et al., 2014); The C: N and C: P ratios in plants indicate the ability of the plant to assimilate and accumulate C and the degree of nutrient utilization, which can be used as an indicator of plant growth rate (Agren, 2004). Soil quality plays a decisive role in plant growth as an important means of obtaining nutrients for plant growth and development. When soil nutrients are insufficient and restrict plant growth, plants will continuously adjust the physiological and ecological processes of various nutrient organs to meet their growth and development needs (Schreeg et al., 2014). Therefore, it is of great significance to analyze the relationship of the ecological stoichiometric characteristics of C, N, and P elements between plants and soil in the ecosystem, to clarify the correlation between plants and soils ecological stoichiometric, to reveal the interaction between C, N and P elements and the relationship between restriction and balance.

The Yellow River Delta Nature Reserve is the youngest and most complete wetland ecosystem in the world (Li et al., 2021). However, due to natural factors and anthropogenic activities in recent years, the water and salt balance of wetland soil in the Yellow River Delta has been destroyed and the degree of soil salinization has increased (Zhao et al., 2022), limiting the growth of many plants in the area, which is a key factor affecting the sustainable development of agriculture. Halophytes are the main vegetation type in this saline-alkali area. According to plants’ different salt tolerance modes, halophytes are divided into three types: salt repellent, salt accumulating, and salt secreting. They all have unique physiological processes of salt tolerance, which can affect the physicochemical properties of soil and the structure of the rhizosphere microbial community by adjusting the salt content of the soil, forming a microenvironment conducive to plant growth and development, it has an important impact on soil improvement of saline-alkali land (Lau and Lennon, 2012; Lareen et al., 2016). At present, most studies on the ecological stoichiometry of halophytes have focused on the changes in the stoichiometric ratio between plants and soil surface ecosystems under different external environments (Wang et al., 2010; Wen et al., 2021), while studies on nutrient organs and soil profile stoichiometry changes and salt accumulation in different types of halophytes are relatively limited.

Therefore, three different types of halophyte communities, *Phragmites communis* (salt repellent), *Suaeda salsa* (salt accumulating), and *Aeluropus sinensis* (salt secreting), were selected in the Yellow River Delta to explore the ecological stoichiometry, salt characteristics, and intrinsic relationships in the plant-soil system, and to comprehensively assess the homeostasis characteristics of nutrient organ stoichiometry of the three halophytes, as well as to reveal the allocation strategies of different types of halophytes for nutrients and salinity in saline habitats from the perspective of the effect of salinity on ecological stoichiometric characteristics. To this end, we have the following three hypotheses: First, the salt tolerance mechanisms of halophytes influence the ecological stoichiometry and salt ion content characteristics at the soil profile level and among plant nutrient organs; Second, the correlation between ecological stoichiometry characteristics and salt ion content of halophytes varies depending on the salt tolerance mechanisms of halophytes; Third, due to the different salt tolerance strategies developed by halophytes as a result of their long-term adaptation to saline soils, their growth-limiting elements as well as their internal stability therefore differ somewhat and show different patterns in the internal stability of their nutrient organs.

## 2 Materials and methods

### 2.1 Study area

**Figure 1:**
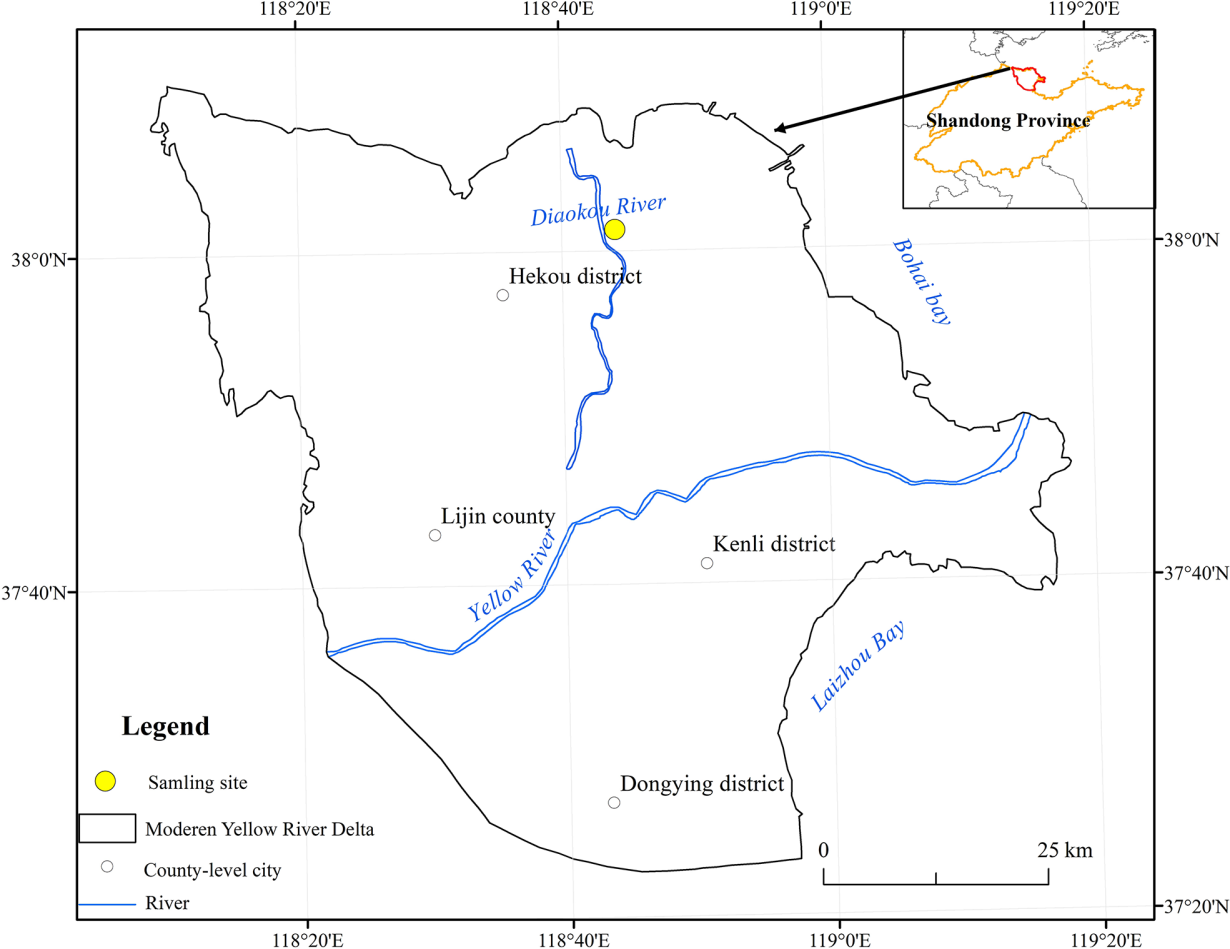
Location of the study area (Zhao et al., 2022).

The study area (N 37°55’26”, E 118°34’37”) is located in Dongying City, Yellow River Delta, as shown in **Figure 1**. The region has a semi-humid continental monsoon climate with four distinct seasons. The annual average temperature is 12.1 °C, and the regional average precipitation is about 550 mm; the terrain is relatively flat, mostly sandy and argillaceous soils, which are easy to compact and with poor nutrient conditions. The vegetation types are mainly *P. communis*, *S. salsa*, *A. sinensis*, and *Tamarix chinensis*. The area was classified as a moderately saline soil area based on the results of soil salinity determination at the above sampling sites and the forestry industry-standard (LY/T2959-2018) released in 2018.

### 2.2 Plant and soil sample collection

Plants: Three fixed sites of 30 m × 30 m were selected in the study area. According to the serpentine distribution method, three small plots of 1 m × 1 m were set in each plot based on the distribution of three types of halophytes. We selected the intact roots, stems, and leaves of three healthy plants in each small sample square, took them back to the laboratory with zip lock bags, and immediately cleaned the soil on the roots, stems, and leaves of the plants until they were free of mud. After drying, removing any impurities such as withered material and rotten roots; placing the clean plant organs in a 105°C oven for 30 min, and then continue drying in a 65°C oven for 2-3 days to achieve a constant weight. The dried plant roots, stems, and leaves were crushed with a plant crusher and ground with a mortar for storage.

Soil: A soil sampling point was set at each plant sampling point, and the soil samples were sampled from three soil layers by soil drill: 0-20 cm, 20-40 cm, and 40-60 cm, 5 drills of soil were taken from each soil layer. After evenly mixing, keep about 1 kg by quartering, placing them in zip lock bags and returning to the laboratory, air-drying them naturally for 10-15 days, and picking out the plant residues. After grinding, they screened through 10 a mesh sieve and a 60 mesh sieve respectively for standby.

### 2.3 Plant and soil sample determination

Plants: TC and TN were determined by an elemental analyzer; TP was determined by the molybdenum antimony anti-colorimetric method; K^+^, Ca^2+^, Na^+^, and Mg^2+^ contents were digested with concentrated sulfuric acid-perchloric acid and determined by an inductively coupled plasma emission spectrometer.

Soil: soil salinity was determined by the residue drying method; pH was determined by the potentiometric method; TC and TN were determined by an elemental analyzer; TP was determined by the molybdenum antimony anti-colorimetric method; K^+^, Ca^2+^, Na^+^, and Mg^2+^ contents were digested with concentrated sulfuric acid-perchloric acid and determined by an inductively coupled plasma emission spectrometer; Cl^-^, SO_4_^2-^, and NO_3_^-^contents were determined by ion chromatography (Bao, 2000).

### 2.4 Comprehensive evaluation method of plant homeostasis

#### 2.4.1 Calculation of homeostasis index

The homeostasis index was calculated using the model (Sterner and Elser, 2002): y=cx^1/H^. In this study, y is the nutrient elements and stoichiometric ratios (C, N, P, C: N, C: P, N: P) of plant roots, stems, and leaves; x is the corresponding nutrient elements and stoichiometric ratios of plant surface soil, c is a constant, and H is the homeostasis index. The homeostasis index H was calculated by the power function index formula in Excel 2016. For the convenience of statistics, this study also uses 1/H to measure the strength of homeostasis (Hood and Sterner, 2010). And classified 1/H into four types: 0 < 1/H < 0.25, steady-state; 0.25 < 1/H < 0.5, weakly steady-state; 0.5 < 1/H < 0.75 weakly sensitive; and 1/H > 0.75, sensitive (Persson et al., 2010).

#### 2.4.2 Comprehensive evaluation method of homeostasis

In this study, the fuzzy mathematics subordinate method was used to evaluate plant homeostasis (Xu et al., 2021). The formula was: X(μ)= $\frac{X-X_{min}}{X_{max}-X_{min}}$ . In the formula, X is the H value of a certain element or stoichiometric ratio of the plant, Xmax is the maximum H value of a certain index of the roots, stems and leaves in the halophyte, and Xmin is the minimum value. Based on calculating the homeostasis index H, the subordinate values of the three halophyte nutrient elements and the stoichiometric ratios were calculated in different organs, and then the subordinate values of each indicator in different halophyte organs were accumulated and averaged. Finally, the subordinate values of each indicator of each plant were accumulated to obtain the average value. The homeostasis of the three halophytes was evaluated comprehensively by comparing the magnitude of the total mean value of the homeostasis subordinate to the halophytes (Sun et al., 2006)

### 2.5 Data analysis

Excel 2016 was used to perform statistics and calculations on the data, and the SPSS 20.0 Duncan (D) method was used to perform ANOVA one-way analysis. The paper’s data were expressed as mean ± standard error, with a significance level of *P* = 0.05. Origin 2019b was used for box and histogram plotting.

## 3 Results

### 3.1 Ecological stoichiometry and salt ions characteristics of different halophytes

#### 3.1.1 Characteristics of TC, TN, and TP content distribution in nutrient organs

**Figure 2:**
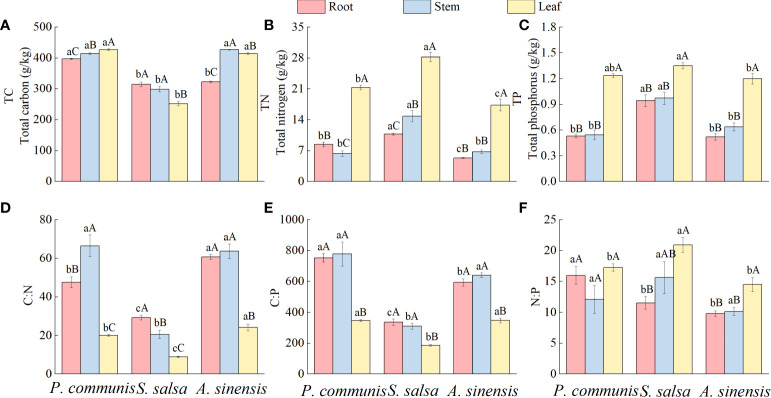
Characteristics of TC **(A)**, TN **(B)** and TP **(C)** contents and C:N **(D)**, C:P **(E)** and N:P **(F)** stoichiometric ratios in halophyte organs. Capital letters indicate the difference between different organs in the same halophyte, and lowercase letters donate the difference of different halophytes of the same organ (*P* < 0.05).

There were great differences in TC content among the three nutrient organs of halophytes (**Figure 2**), with the *P. communis* showing: leaf > stem > root, the *S. salsa*: root = stem > leaf, and the *A. sinensis*: stem > leaf > root. TN and TP contents in leaves were significantly higher than those in roots and stems in the three halophytes. *P. communis* stems had significantly lower TN content than roots, while *S. salsa* and *A. sinensis* stems had significantly higher TN content than roots (*P* < 0.05).

Comparing the same nutrient organs of different plants, it was found that the TC content of *P. communis* roots was significantly higher than that of *S. salsa* and *A. sinensis*, and the TC content of *P. communis* and *A. sinensis* stems and leaves was significantly higher than that of *S. salsa* (*P* < 0.05). Among the different halophytes, roots and leaves TN content showed as follows: *S. salsa* > *P. communis* > *A. sinensis*, stem: *S. salsa* > *P. communis* = *A. sinensis*; the TP content of *S. salsa* roots and stems was significantly higher than that of *P. communis* and *A. sinensis*, and the TP content of leaves was significantly higher than that of *A. sinensis* (*P* < 0.05).

#### 3.1.2 Eco-stoichiometric ratio characteristics of nutrient organs

Significant differences in C: N among different organs of the same halophytes (**Figure 2**), *P. communis*: stem > root > leaf, *S. salsa*: root > stem > leaf, *A. sinensis*: root = stem > leaf; C: P content of the roots and stems in the three halophytes was significantly higher than that of the leaves; N: P was significantly higher in *S. salsa* leaves than in roots, and in *A. sinensis* leaves than in roots and stems (*P* < 0.05), but there was no significant difference in N: P in different organs of *P. communis*(*P* > 0.05).

The differences in root and leaf C: N among different halophytes were as follows: *A. sinensis* > *P. communis* > *S. salsa*, stem: *A. sinensis* = *P. communis* > *S. salsa*; C: P difference in stems and leaves was: *P. communis* = *A. sinensis* > *S. salsa*, root: *P. communis* > *A. sinensis* > *S. salsa*; N: P in roots of three halophytes was: *P. communis* > *S. salsa* = *A. sinensis*, leaf: *S. salsa* > *P. communis* = *A. sinensis*, and there was no significant difference in N: P between stems.

#### 3.1.3 Total cation content of nutrient organs of halophytes

**Figure 3:**
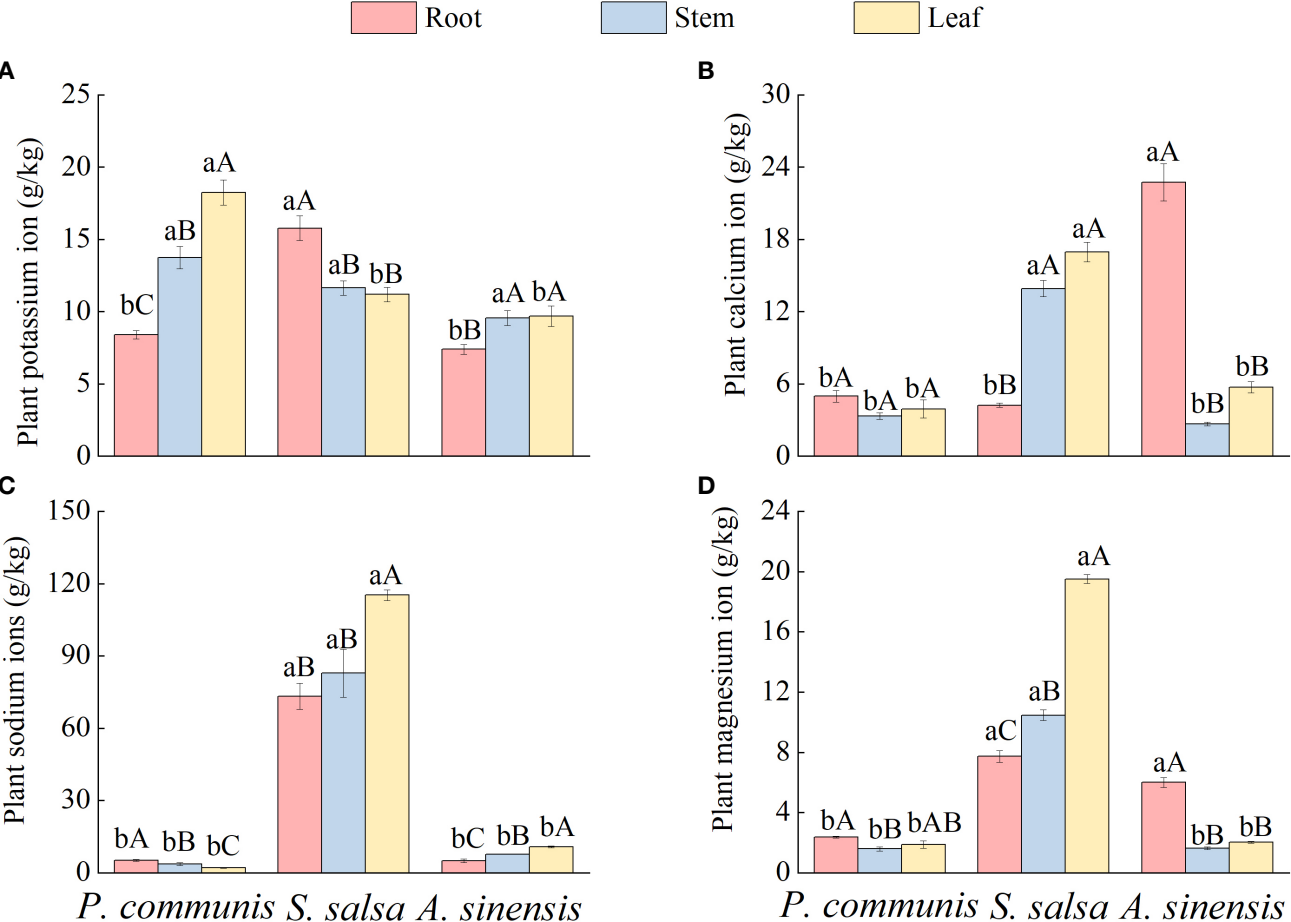
K+ **(A)**, Ca2+ **(B)**, Na+ **(C)**, Mg2+ **(D)** cation content in organs of halophyte. Capital letters indicate the difference between different organs in the same halophyte, and lowercase letters donate the difference of different halophytes of the same organ (P < 0.05).

The different ion contents of plant roots, stems, and leaves varied under the same halophyte type (**Figure 3**), where the variability of K^+^ content in *P. communis* nutrient organs showed: leaf > stem > root, *S. salsa*: root > stem = leaf, *A. sinensis*: stem = leaf > root; The Ca^2+^ content was significantly different among the three organs of *S. salsa* and *A. sinensis* (*P* < 0.05), *S. salsa*: stem = leaf > root, *A. sinensis*: root > stem = leaf, and no significant difference in *P. communis*; the difference in Na^+^ content of different organs of *P. communis* was: root > stem > leaf, while *A. sinensis* was the opposite: root < stem < leaf, and *S. salsa*: leaf > root = stem; The differences in Mg^2+^ content of nutrient organs of *P. communis* were as follows: root > stem, *S. salsa*: leaf > stem > root, and *A. sinensis*: leaf > stem = root.

Comparing the same nutrient organs of different halophytes (**Figure 3**), it was found that the K^+^ and Na^+^ contents in the roots of *S. salsa*, Ca^2+^, Na^+^, and Mg^2+^ contents of stems and leaves were significantly higher than those in *P. communis* and *A. sinensis*. In addition, the K^+^ contents of *P. communis* leaves were significantly higher than those of *S. salsa* and *A. sinensis* (*P* < 0.05), and the Ca^2+^ and Mg^2+^ contents of *A. sinensis* roots were significantly higher than those of *P. communis* (*P* < 0.05).

#### 3.1.4 Correlation between ecological stoichiometry and cations in halophytes

**Figure 4:**
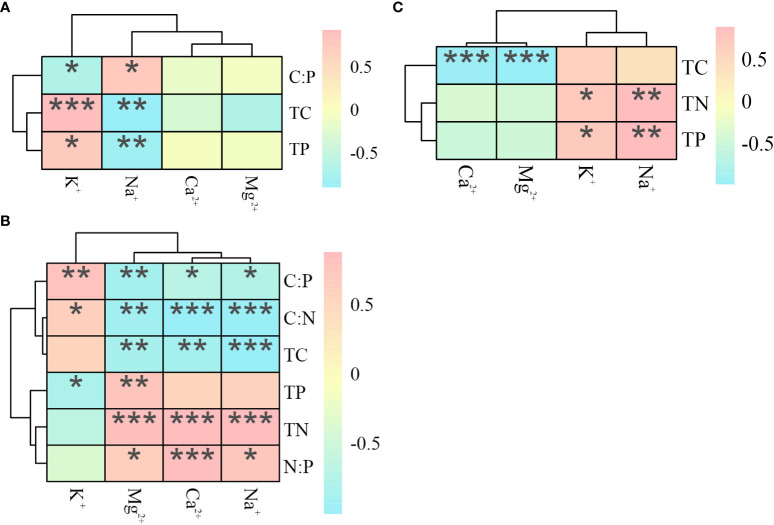
Heat map of the correlation between ecological stoichiometry and cations in *P. communis* **(A)**, *S. salsa* **(B)** and *A. sinensis* **(C)**. *, ** and *** represent significant differences at 0.05, 0.01 and 0.001 levels respectively.

In the salt repellent plant *P. communis* (**Figure 4**), both TC and TP were positively correlated with K^+^ content and negatively correlated with Na^+^, contrary to the above results, C: P was negatively correlated with K^+^ content and positively correlated with Na^+^ (*P* < 0.05). In *S. salsa*, TC, C: N, and C: P were negatively correlated with the contents of Ca^2+^, Na^+^, and Mg^2+^, and TN and N: P were significantly positively correlated with the contents of the above ions, in addition, C: N and C: P were positively correlated with K^+^ content, TP was negatively correlated with K^+^ content, and positively correlated with Mg^2+^. The *A. sinensis* TN and TP were significantly positively correlated with the K^+^ and Na^+^ content, and the TC was negatively correlated with the Ca^2+^ and Mg^2+^ content.

### 3.2 Ecological stoichiometry and salinity characteristics of different halophyte soils

#### 3.2.1 Distribution characteristics of TC, TN, and TP content in halophyte soil at different soil depths

**Figure 5:**
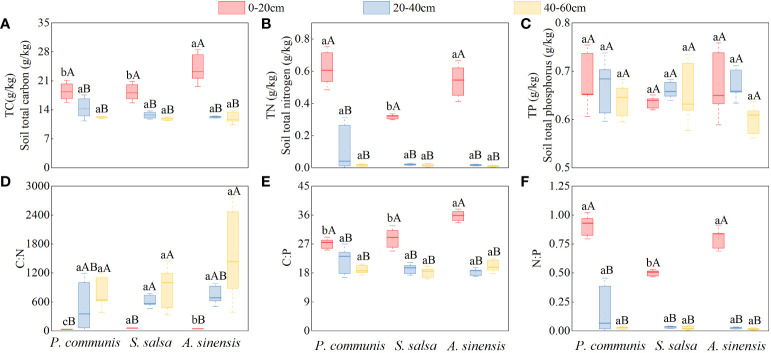
Halophytes characteristics of TC **(A)**, TN **(B)** and TP **(C)** contents and C:N **(D)**, C:P **(E)** and N:P **(F)** stoichiometric ratios in soils of different soil layers. Capital letters indicate the difference among different soil layers in the same halophyte, lowercase letters indicate the difference among different halophytes in the same soil layer (*P* < 0.05).

The TC and TN contents of the three halophytes in the 0-20 cm soil layer were significantly higher than those of the respective 20-40 cm and 40-60 cm soil layers (*P* < 0.05), and the TP contents of the soils were not significantly different (*P* > 0.05) under different soil layers of the same halophytes (**Figure 5**).

Comparing different halophytes in the same soil layer, it was found that only the contents of TC and TN showed differences in the 0-20 cm soil layer (*P* < 0.05). TC: *A. sinensis* > *P. communis* = *S. salsa*, TN: *P. communis* = *A. sinensis* > *S. salsa*, while the TC and TN contents of three different halophytes were not significantly different in 20-40 cm and 40-60 cm soil layers and TP in three soil layers. (*P* > 0.05).

#### 3.2.2 Ecological stoichiometric ratio characteristics of halophyte soil at different soil depths

The C: N of *P. communis* and *A. sinensis* 0-20 cm soil layer was significantly higher than that of their respective 40-60 cm soil layers (**Figure 5**), and the C: N of S. salsa 0-20 cm soil layer was significantly higher than that of the 20-40 cm and 40-60 cm soil layers (*P* < 0.05); the C: P and N: P contents of the three halophytes 0-20 cm soil layer were significantly higher than that of their respective 20-40 cm and 40-60 cm soils (*P* < 0.05).

Comparing different halophytes in the same soil layer, it was found that the soil ecological stoichiometric ratios of the three halophytes only varied significantly before the 0-20 cm soil layer, the variability of C: N values was shown by: *S. salsa* > *A. sinensis* > *P. communis*; the C: P content of the *A. sinensis* soil was significantly higher than that of *P. communis* and *S. salsa* (*P* < 0.05), and the N: P content of *P. communis* and *A. sinensis* soil was significantly higher than that of *S. salsa* (*P* < 0.05). The C: N, C: P, and N: P contents of the three halophyte soils were not significantly different in the 20-40 cm and 40-60 cm soil layers (*P* > 0.05).

#### 3.2.3 Distribution characteristics of salt content in halophyte soil at different soil depths

**Figure 6:**
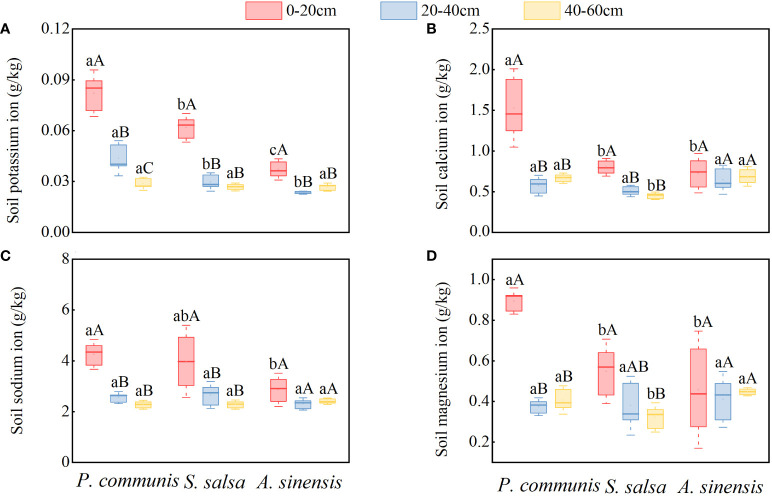
K+ **(A)**, Ca2+ **(B)**, Na+ **(C)**, Mg2+ **(D)** cation content in soils of halophyte. Capital letters indicate the difference between different organs in the same halophyte, and lowercase letters donate the difference of different halophytes of the same organ (P < 0.05).

As shown in **Figure 6**, the K^+^, Ca^2+^, and Na^+^ contents of *P. communis* and *S. salsa* 0-20 cm soil layers were significantly higher (*P*<0.05) than 20-40 cm and 40-60 cm soil layers, and the Mg^2+^ contents were significantly higher than 40-60 cm soil layers for the same halophyte in different soil layers, while only the K^+^ contents of *A. sinensis* 0-20 cm soil layers were significantly higher than the 40-60 cm soil layer.

Comparing the cation contents of different halophytes in the same soil layer (**Figures 6**), it was found that the K^+^, Ca^2+^, and Mg^2+^ contents of *P. communis* soil were significantly higher than those of *S. salsa* and *A. sinensis* in the 0-20 cm soil layer, and the Na^+^ content was only significantly higher than that of *A. sinensis*. The Ca^2+^ and Mg^2+^ contents in the 40-60 cm soil layer were significantly higher in *P. communis* and *A. sinensis* than in *S. salsa* (*P* < 0.05).

**Figure 7:**
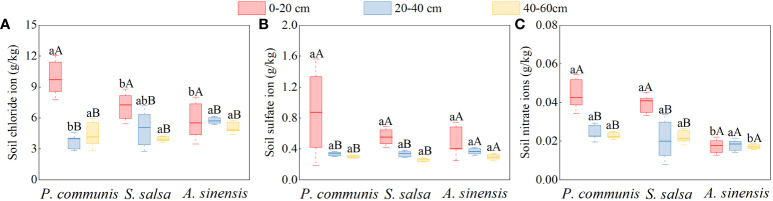
Cl- **(A)**, SO42- **(B)**, NO3- **(C)** anion content in soils of halophyte. Capital letters indicate the difference between different organs in the same halophyte, and lowercase letters donate the difference of different halophytes of the same organ (P < 0.05).

As shown in **Figure 7**, the Cl^-^, SO_4_^2-^, and NO_3_^-^ contents in the 0-20 cm soil layers of *P. communis* and *S. salsa* were significantly higher than those of their respective 20-40 cm and 40-60 cm soil layers (*P* < 0.05), and the Cl^-^ content of the 0-20 cm soil layer of *A. sinensis* was significantly higher than that of other soil layers (*P* < 0.05). When comparing the soil anion contents of different halophytes in the same soil layer, we discovered that the Cl^-^ and NO_3_^-^ contents of *P. communis* 0-20 cm soil layer were significantly higher than that of *A. sinensis*, and the Cl^-^ content was significantly higher than that of *S. salsa*. The Cl^-^ content of *P. communis* in the 20-40 cm soil layer was significantly lower than that of *A. sinensis*; in the 40-60 cm soil layer, the NO_3_^-^ content of *P. communis* and *S. salsa* soil was significantly higher than that of *A. sinensis* (*P* < 0.05), while SO_4_^2-^ did not differ significantly among the three halophytes in the same soil layer.

#### 3.2.4 Correlation between soil ecological stoichiometric characteristics and soil salinity factors

**Figure 8:**
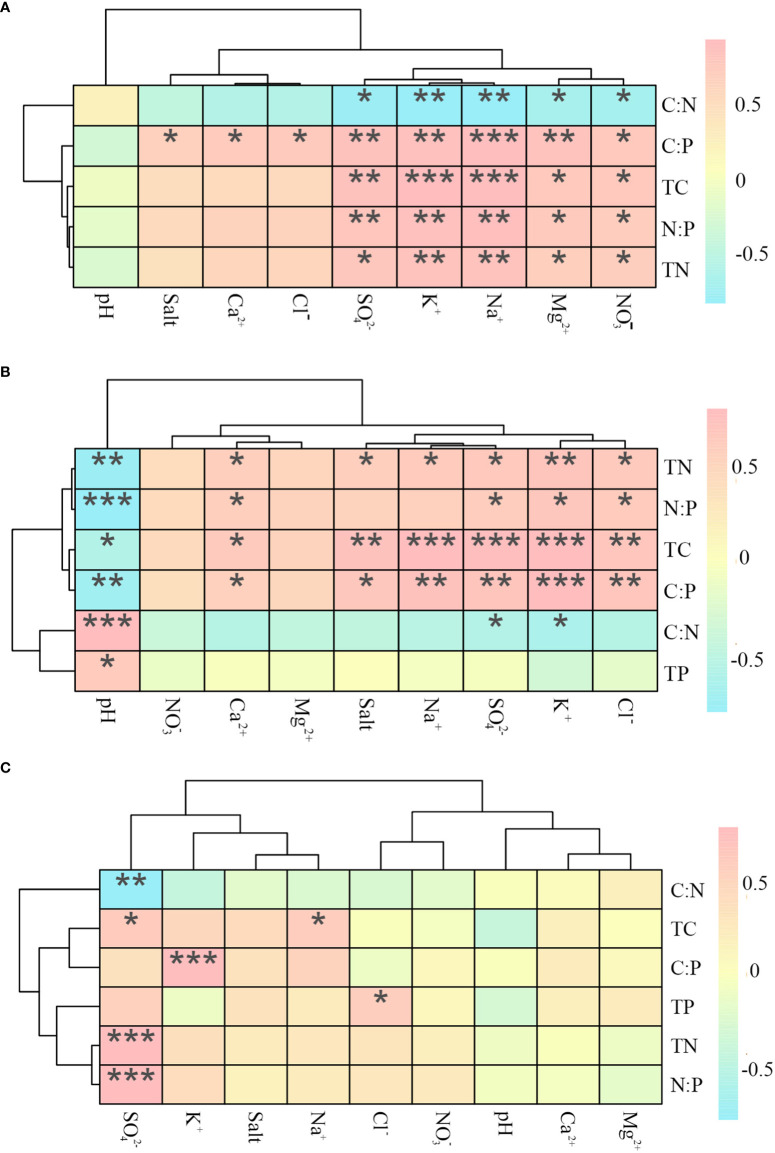
Heat map of correlations between ecological stoichiometry and ions in *P. communis* **(A)**, *S. salsa* **(B)** and *A. sinensis* **(C)**. *, ** and *** represent significant differences at 0.05, 0.01 and 0.001 levels respectively.

The TC, TN, C: P, and N: P values of *P. communis* soil were positively correlated with the contents of K^+^, Na^+^, Mg^2+^, SO_4_^2-^, and NO_3_^-^ in the soil, while the C: N was negatively correlated with the above ions, where C: P also showed positive correlations with Ca^2+^, Cl^-^, and soil salinity (**Figures 8**).

*S. salsa* soil TC, TN, and C: P values were positively correlated with K^+^, Ca^2+^, Na^+^, Cl^-^, SO_4_^2-^ and salt content, negatively correlated with pH; N: P was positively correlated with K^+^, Ca^2+^, Cl^-^, and SO_4_^2-^ ions content and negatively correlated with pH; C: N was negatively correlated with K^+^ and SO_4_^2-^ contents, while TP was positively correlated with pH.

In *A. sinensis* soil, the values of TC, TN, and N: P were positively correlated with SO_4_^2-^ content, where TC was also positively correlated with Na^+^ content; C: P was positively correlated with K^+^ content; TP was positively correlated with Cl^-^ content, and C: N was negatively correlated with SO_4_^2-^ content.

### 3.3 Correlation between plant ecological stoichiometry and soil factors

#### 3.3.1 Correlation between plant ecological stoichiometry and soil ecological stoichiometry

*P. communis* plant TC was negatively correlated with soil TC, TN, C: P, N: P, and positively correlated with C: N value (**Table 1**); plant TP was negatively correlated with soil TC.

**Table 1:** Correlation between plant and soil ecological stoichiometry of three different halophytes.

Plant types		SoilTC	SoilTN	SoilTP	SoilC: N	SoilC: P	SoilN: P
*P. communis*	Plant TC	-0.92***	-0.85**	-0.48	0.85**	-0.83**	-0.83**
	Plant TN	-0.4	-0.23	-0.25	0.23	-0.17	-0.22
	Plant TP	-0.7*	-0.5	-0.62	0.50	-0.37	-0.48
	Plant C: N	0.4	0.23	0.25	-0.23	0.17	0.22
	Plant C: P	0.62	0.42	0.62	-0.42	0.28	0.40
	Plant N: P	-0.02	0.05	0.20	-0.05	-0.07	0.03
*S. salsa*	Plant TC	0.77*	0.83**	-0.07	-0.72*	0.75*	0.78*
	Plant TN	-0.92***	-0.87**	0.22	0.77*	-0.88**	-0.82**
	Plant TP	-0.7*	-0.52	-0.1	0.57	-0.62	-0.47
	Plant C: N	0.87**	0.92***	-0.25	-0.83**	0.87**	0.88**
	Plant C: P	0.7*	0.63	0	-0.7*	0.65	0.62
	Plant N: P	-0.73*	-0.75*	0.22	0.62	-0.7*	-0.7*
*A.sinensis*	Plant TC	-0.55	-0.37	0.18	0.4	-0.82**	-0.38
	Plant TN	-0.68*	-0.92***	-0.52	0.92***	-0.42	-0.88**
	Plant TP	-0.77*	-0.88**	-0.73*	0.78*	-0.43	-0.8**
	Plant C: N	0.28	0.62	0.52	-0.63	-0.18	0.57
	Plant C: P	0.23	0.48	0.92***	-0.32	-0.37	0.35
	Plant N: P	-0.38	-0.72*	-0.43	0.73*	-0.02	-0.7*

*, ** and *** showed significance at 0.05, 0.01 and 0.001 levels respectively.

Plants TC and C: N values in *S. salsa* were positively correlated with soil TC, TN, C: P, N: P, and negatively correlated with soil C: N; plant TN, N: P were negatively correlated with soil TC, TN, C: P, N: P, and plant TN was also positively correlated with soil C: N; plant TP was negatively correlated with soil TC; plant C: P was negatively correlated with soil TC, and negatively correlated with C: N.

The plant TC content of *A. sinensis* was negatively correlated with soil C: P; plant TN and TP were negatively correlated with soil TC, TN, and N:P, and positively correlated with soil C: N. Among them, plant TP was negatively correlated with soil TP; plant C: P was positively correlated with soil TP; plant N: P was negatively correlated with soil TN and N:P, and positively correlated with soil C: N.

#### 3.3.2 Correlation between plant ecological stoichiometry and soil salinity factor

Compared to the correlations between the three halophytes’ ecological stoichiometry and soil salt ion content (**Table 2**), it was found that the *P. communis* plants’ TC content was negatively correlated with K^+^, Na^+^, SO_4_^2-^, and NO_3_^-^. *S. salsa* plant TC was positively correlated with soil K^+^, Ca^2+^, SO_4_^2-^ and salinity; plant TN and TP were negatively correlated with the content of Ca^2+^, Na^+^, SO_4_^2-^ and salinity. In addition, plant TN was positively correlated with soil pH and negatively correlated with soil K^+^ content; C: N and C: P were positively correlated with soil Ca^2+^, Na^+^, Cl^-^, SO_4_^2-^ and salinity, among them, C: N was also positively correlated with soil K^+^ content, and negatively correlated with soil pH; N: P was negatively correlated with soil K^+^, and NO_3_^-^ content. In *A. sinensis*, the plant TC was negatively correlated with soil K^+^; the plant TN and TP contents were negatively correlated with SO_4_^2-^.

**Table 2:** Correlation between organ ecological stoichiometry and soil salt ions in three halophytes.

Plant types		pH	salinity	K^+^	Ca^2+^	Na^+^	Mg^2+^	Cl^-^	SO_4_^2-^	NO_3_^-^
*P. communis*	Plant TC	0.29	-0.52	-0.93***	-0.52	-0.87**	-0.62	-0.62	-0.95***	-0.88**
	Plant TN	0.08	-0.25	-0.47	0.3	-0.38	0.1	0.17	-0.43	-0.35
	Plant TP	0.03	-0.12	-0.65	0.02	-0.5	-0.08	-0.02	-0.5	-0.4
	Plant C: N	-0.08	0.25	0.47	-0.3	0.38	-0.1	-0.17	0.43	0.35
	Plant C: P	-0.08	0.15	0.58	-0.22	0.4	-0.08	-0.15	0.43	0.35
	Plant N: P	0.19	-0.53	-0.25	0.12	-0.25	-0.03	-0.03	-0.33	-0.32
*S. salsa*	Plant TC	-0.65	0.73*	0.77*	0.7*	0.67	0.53	0.67	0.82**	0.35
	Plant TN	0.75*	-0.78*	-0.93***	-0.78*	-0.78*	-0.6	-0.68	-0.9***	-0.58
	Plant TP	0.4	-0.78*	-0.6	-0.72*	-0.73*	-0.6	-0.68	-0.82**	-0.18
	Plant C: N	-0.78*	0.77*	0.88**	0.8**	0.75*	0.63	0.7*	0.88**	0.57
	Plant C: P	-0.53	0.8**	0.63	0.78*	0.73*	0.67	0.73*	0.85**	0.27
	Plant N: P	0.63	-0.5	-0.78*	-0.62	-0.53	-0.42	-0.37	-0.67	-0.72*
*A.sinensis*	Plant TC	-0.37	-0.45	-0.88**	-0.23	-0.65	-0.12	0.37	-0.25	0.22
	Plant TN	0.02	-0.27	-0.38	0.13	-0.33	0.23	-0.08	-0.8**	0.05
	Plant TP	0.08	-0.6	-0.47	-0.27	-0.63	-0.18	-0.3	-0.78*	0.08
	Plant C: N	-0.23	-0.07	-0.27	-0.43	-0.13	-0.45	0.08	0.53	-0.12
	Plant C: P	-0.32	0.33	-0.3	0.15	0.17	0.2	0.53	0.47	-0.05
	Plant N: P	0.18	0.13	0	0.45	0.1	0.57	0.02	-0.7*	-0.1

*, ** and *** showed significance at 0.05, 0.01 and 0.001 levels respectively.

### 3.4 Homeostasis characteristics of different types of halophytes

**Figure 9:**
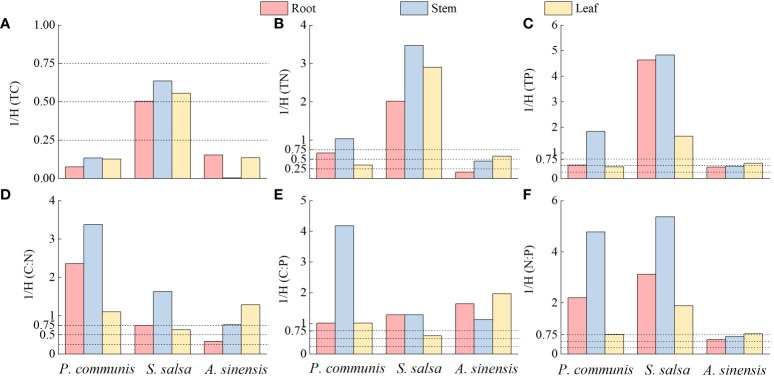
Ecological stoichiometric homeostasis indices of TC **(A)**, TN **(B)**, TP **(C)**, C:N **(D)**, C:P **(E)** and N:P **(F)** in three halophytes.

The analysis of the ecological stoichiometry homeostasis of different organs in different halophytes according to the four type intervals delineated by Persson et al. (2010) showed (**Figure 9**) that the C elements of *P. communis* and *A. sinensis* roots, stems, and leaves were stable, but the C elements of three nutrient organs in *S. salsa* were weakly sensitive; the N and P elements of *P. communis* roots were weakly sensitive, stems were sensitive and leaves were weakly stable, and the N and P elements of *S. salsa* roots, stems and leaves were sensitive, the N elements of *A. sinensis* roots were stable and P elements were weakly stable, the N and P elements of stems were weakly stable and leaves were weakly sensitive. Collectively, the homeostasis of C, N and P elements in three halophytes was: C > N > P.

C: N, C: P, and N: P of *P. communis* roots, stems, and leaves were sensitive; C: N and C: P of *S. salsa* roots and stems were sensitive, leaves were weakly sensitive, and N:P of three organs were sensitive; C: N of *A. sinensis* stems and leaves were sensitive, roots were weakly stable, and C: P of three organs were sensitive, N: P of roots and stems were weakly sensitive, and leaves were sensitive.

The fuzzy mathematics subordinate method was used to comprehensively evaluate the C, N, and P nutrient contents and the C: N, C: P, and N: P ecological stoichiometric indices of three halophytes (**Table 3**), and obtained three overall means of subordinate functions for halophytes. It was found that the total average values of *A. sinensis*, *P. communis*, and *S. salsa* were 0.75, 0.46, and 0.22, indicating that the salt secreting plant *A. sinensis* had the strongest homeostasis and the salt accumulating plant *S. salsa* had the weakest homeostasis.

**Table 3:** Fuzzy mathematics subordinate value and homeostasis evaluation of three halophytes.

Halophyte	TC	TN	TP	C: N	C: P	N: P	Average	Rank
*P. communis*	0.68	0.51	0.68	0.05	0.47	0.37	0.46	2
*S. salsa*	0	0	0	0.56	0.76	0	0.22	3
*A. sinensis*	0.77	0.85	0.89	0.67	0.33	0.99	0.75	1

## 4 Discussion

### 4.1 Salt tolerance characteristics of different halophytes

Relevant studies have found that halophytes adapt to long-term saline-alkali stress mainly by accumulating inorganic ions in the plant, and the salt ion content in the plant gradually increases during transport from the roots to the tip, forming a “salt island” effect (Yong et al., 2016). In our study, the distribution pattern of the total cation content of the salt repellent plant *P. communis* and the salt accumulating plant *S. salsa* was consistent with this, i.e., root < stem < leaf. However, the distribution of salt ion content in *A. sinensis* and their soils showed different patterns, presumably related to the unique salt tolerance strategy of salt secreting: salt glands or salt vesicles are generally present in salt secreting, which can absorb salt ions from the soil through the roots and excrete excess soluble salt ions through salt glands or salt vesicles (Fu et al., 2013), resulting in a high salt ion content in the roots and a low salt ion content in the soil.

Na^+^ is an important inorganic osmoregulatory substance, Parida and Das (2005) have pointed out that one of the mechanisms of salt tolerance in plants is to control the uptake of Na^+^ by roots and reduce its translocation to the leaves. The results of this study showed that the salt repellent plant *P. communis* root will limit Na^+^ transport to the ground, reduce its translocation to stems and leaves, translocate plant Na^+^ to the soil, and balance the osmotic pressure in the plant by absorbing more K^+^, fundamentally reducing the toxicity of salt ions to ensure *P. communis* growth in salt-stressed environments, which is consistent with the results of Peng et al. (2017). In addition, the cations in the *S. salsa* accumulated in the leaves of the plants and showed stronger uptake of Na^+^ and Mg^2+^ than the other two halophytes. This is because *S. salsa* is a typical salt accumulating plant that can absorb salt from the soil to accumulate in its fleshy stems and leaves (Jia et al., 2021). The accumulation of Na^+^ in *S. salsa* leaves is the main reason for leaf succulence, which in turn increases the number of cells in the plant, allowing more Na^+^ to enter the vesicles, thereby reducing the osmotic stress caused by Na^+^ to the plant, which is conducive to the survival of *S. salsa* in saline soil (Wang et al., 2001).

In the present study, the anion and cation contents of the soils of the salt repellent plant *P. communis* and the salt accumulating plant *S. salsa* were similar in vertical structure, i.e., the salt was concentrated in the top soil layer, in agreement with the view proposed by Su et al. (2022), which is because soil salts mainly originate from groundwater, and under the absorption and transpiration of plants, the salinity in the lower soil migrates to the upper layer, the salinity of the soil surface was higher (Ali et al., 2022). However, there are relatively few ionic species that differ in the vertical structure of the salt secreting plant *A. sinensis* soil, mainly K^+^ and Cl^-^, which differs from that of Zhang et al. (2016) for the salt secreting plant *T. chinensis*, and the reason for this phenomenon may be related to the depth of the root system of a halophyte, which needs to be further investigated.

### 4.2 Ecological stoichiometric characteristics of three halophyte organs

The C, N, and P contents in plant organs are one of the important indicators of the nutritional status of plants, which can reflect the adaptive defense responses to the external environment and the growth status of plants (Shi et al., 2021). In our study, we found that the three nutrient elements differed significantly among halophytes and in different organs of the same halophyte, and the results were consistent with the first hypothesis to some extent, which is keep up with the findings of Wen et al. (2021) on different vegetation types in semi-arid regions, Yang et al. (2019) on typical vegetation in temperate deserts, and Zeng et al. (2016) on different vegetation communities in the Loess Plateau. The main reason for this phenomenon is that the different N and P requirements of halophytes affect their photosynthetic and C allocation abilities, resulting in different nutrient contents and ratios among different halophytes (Zechmeister et al., 2015). However, the nutrient changes among roots, stems, and leaves of different types of halophytes showed consistency again during plant growth. For example, three different types of halophytes showed higher N and P contents in their leaves, which is consistent with most studies (Zhang et al., 2021a; Zhang et al., 2021b). On the one hand, this is because the sampling time is summer when the transpiration of leaves is vigorous, and the N and P absorbed by roots are transported upward to the leaves with water, resulting in the enrichment of N and P in the leaves. On the other hand, it is because the plants are in the growth period when the leaves are photosynthetically active and require large amounts of N and P elements to synthesize chlorophyll, proteins, and nucleic acids (Liu et al., 2015). In addition, the higher degree of root and stem fibrillation in all three halophytes resulted in significantly higher C: N values than their respective leaves, which is consistent with the findings of Shi et al. (2021).

Zeng et al. (2015) found that plant leaf C: N and C: P values were proportional to the nutrient use efficiency of plant N and P elements and inversely proportional to plant growth rate, it is clear that the growth rate of *S. salsa* among the three halophytes was relatively faster during this period. In addition, the N and P content of leaves were able to determine the plant nutrient limitation in that environment (Zhang et al., 2019), plant growth was limited by N elements when N: P < 14, by N and P elements when 14 < N: P < 16, and by P elements when N: P > 16. Therefore, the growth of *P. communis* and *S. salsa* is mainly limited by P, *A. sinensis* is limited by both N and P, which is consistent to some extent with the finding of Han et al. (Han et al., 2011) that P is the growth-limiting nutrient element for terrestrial plants in China, and the reason why salt plants are limited by P is mainly related to the availability of soil P. Because the Yellow River delta region is subject to seawater erosion, the soil is heavily dominated by the calcium adsorbed form of P, which is more stable and difficult to be absorbed and used by plants (Bian et al., 2022).

### 4.3 Ecological stoichiometric characteristics of three halophytes in different soil layers

The nutrients required by plants to maintain their growth and development are mainly derived from the soil, therefore the soil texture condition has a great influence on plant growth and development. According to Dise et al. (1998), soil texture, nutrient supply capacity, and the decomposition and transformation of nutrient elements by soil microorganisms can be expressed by the stoichiometric characteristics of soil C, N, and P. In our study, the C and N contents of the surface soils of the three different types of halophytes differed from those of other soil layers, but there was no significant variation in P content, which is consistent with the results of Li et al. (2021). The higher C and N content of the soil surface layer is on the one hand due to the return of vegetation residues to the surface soil, high microbial activity, and rapid decomposition rate of dead fallen matter, therefore higher soil nutrient content; on the other hand, it is caused by the C and N reabsorption and transport of the lower soil by the plant roots (Wang and Zheng, 2018). Because the study area is virtually unaffected by anthropogenic tillage activities and because soil P content is mainly associated with the return of soil parent material and plant and animal residues, which are formed over a long period and are difficult to migrate in the soil, P content does not vary significantly in the soil profile (Bian et al., 2022).

Soil C: N values are inversely proportional to soil nutrient cycling rates, and higher C: N values are not conducive to soil organic matter mineralization decomposition and soil organic carbon accumulation (Haney et al., 2004). The present results indicate that the decomposition rate of organic matter is inversely proportional to soil depth, which is consistent with the results of Wang et al. (2021). In addition, soil C: P values were inversely related to soil P effectiveness. Compare to the analysis of soil C: P values in the three halophytes, it is clear that the surface soil P effectiveness of the three halophytes is lower, and the P effectiveness of *P. communis* and *S. salsa* surface soil was higher than that of *A. sinensis*. Plants grow more easily in lower N: P soils (Chen et al., 2009), indicating that deeper soils are more suitable for plant growth, which is because deeper soils are less affected by anthropogenic activities and climatic conditions. Also comparing the N: P values of the surface soil of the three halophytes shows that the *S. salsa* surface soil is more favorable for plant growth than the *P. communis* and *A. sinensis* surface soil. This suggests that the *S. salsa* plant community has certain advantages in improving the surface layer.

### 4.4 Interrelationship between ecological stoichiometric characteristics and salt ion content

Changes in nutrient elements within the organs of halophytes inevitably affect the transport processes of salt ions (Gupta et al., 2020), and studies have shown that the nutrient element content of halophyte soils can affect the N and P nutrient status within the halophytes, and to a certain extent, it affects the accumulation of total salt in halophytes (Wei et al., 2021). In our study, the interrelationship between ecological stoichiometric characteristics and salt ion content of the three halophytes did not show a consistent pattern, in line with the second hypothesis in the previous paper. The nutrient elements as well as stoichiometric ratios of salt repellent plant *P. communis* soil were mainly correlated with the plant TC content, based on the results of the study, it is clear that under suitable soil nutrient conditions, *P. communis* have enhanced photosynthesis and faster physiological metabolic processes, which promote more Na^+^ excretion from plant organs and K^+^ absorption from outside to balance osmotic pressure, while the faster metabolic rate of *P. communis* in turn reduces the content of various salt ions in the soil, creating soil conditions more suitable for *P. communis* growth and development.

Due to the different physiological strategies of salt tolerance developed in the process of adaptation to soil salinization in *S. salsa* and *P. communis*, the correlation between TC content and soil indicators in both showed opposite patterns. By analyzing the TN content of *S. salsa* with the salt ion content in the plants and the salt content in the soil, we found that N and salt ions in halophytes have a mutually reinforcing effect, which is consistent with the results of Yong et al. (2016) on the stoichiometry and salt ions of nutrient organs in four halophytes. In addition, it was found that the higher N content in *S. salsa* plants in soils with lower C and N nutrient content could more effectively promote the uptake of salt ions in the soil by halophytes, which is due to the higher NO_3_^-^ storage capacity of *S. salsa* under low N conditions, it can better maintain N metabolism and photosynthetic performance in the plants (Liu et al., 2017), the results provide some theoretical basis for saline soil improvement.

Unlike *P. communis* and *S. salsa*, soil nutrients and stoichiometric ratios of the salt secreting plant, *A. sinensis*, had significant effects on TP content within *A. sinensis*, while no significant correlation existed between soil factors and plant TP content for both the salt repellent plant, *P. communis*, and the salt accumulating plant, *S. salsa*. The phenomenon suggests that *A. sinensis* improves the ability of this halophyte to adapt to external environmental conditions by accumulating more P elements in nutrient-depleted soils compared to *P. communis* and *S. salsa*.

### 4.5 Evaluation of plant homeostasis

Plants need to maintain relatively stable nutrient ratios (stoichiometric homeostasis) in their bodies for maintaining optimal plant growth levels (Spohn, 2016), and the level of plant stoichiometric homeostasis is influenced by factors such as uptake, transport, distribution, utilization, and release processes of chemical elements in halophyte (Sistla et al., 2015). In our study, the different organs’ stoichiometric homeostasis of the three halophytes did not show a consistent pattern, which is consistent with the third hypothesis, but overall, the homeostasis of C, N, and P elements in halophytes was: C > N > P, which was consistent with the results of previous related studies that the homeostasis of massive elements in plants is higher than trace elements and trace elements are higher than non-essential elements (Han et al., 2011). It was found that plants with higher homeostasis were more conservative in nutrient use and could maintain slow growth in poor environments, while plants with lower homeostasis were more adaptable to external soil changes (Persson et al., 2010). A comprehensive evaluation of plant homeostasis through the fuzzy mathematics subordinate method of halophytes showed that *A. sinensis* had the most homeostasis and *S. salsa* had the least homeostasis, which indicated that *A. sinensis* was more adapted to grow in stable environments and *S. salsa* was more advantageous in variable environments compared to the three halophytes. It is speculated that in the future, as the environmental conditions of saline soils gradually improve, *S. salsa* can become the dominant species for saline soil improvement in the region, therefore, it is recommended to plant *S. Salsa* in areas where saline improvement is being carried out and *A. sinensis* in saline areas with more stable soil conditions. Overall, this study provides some theoretical basis for further discussion of ecological adaptation processes, nutrient allocation and utilization strategies, and plant stoichiometry homeostasis characteristics of halophytes in saline areas of the Yellow River Delta.

## 5 Conclusion

In this area, the salt ion content in the salt repelling plant *P. communis* and the salt accumulating plant *S. salsa* gradually increases during transport from the roots to the tip, forming a salt island effect, and the leaves of *S. salsa* show an extremely strong absorption capacity for Na^+^. The N and P elements were enriched in the leaves of the three halophytes, and the C: N and C: P values of *P. communis* and *A. sinensis* leaves were significantly greater than those of *S. salsa*, indicating that the growth rate of *S. salsa* was relatively faster in this period. Based on the N: P values of the halophytes, it is clear that the growth of *P. communis* and *S. salsa* in this area is mainly limited by P, and that *A. sinensis* is limited by both N and P. The contents of C, N, C: P, and N: P in the surface soil (0-20 cm) of the three halophytes were significantly higher than those in the 20-40 cm and 40-60 cm soil layers, indicating that the deeper soils are more suitable for plant growth. The C:P was lower on the surface soil of *P. communis* and *S. salsa*, and N:P was lower in the surface soil of *S. salsa* than in *P. communis* and *A. sinensis*, indicating that the P effectiveness of the surface soil in *P. communis* and *S. salsa* was high, and the surface soil of *S. salsa* was more favorable for plant growth than in the surface soil of *P. communis* and *A. sinensis*. In contrast to *P. communis* and *S. salsa*, *A. sinensis* can improve the ability of halophytes to adapt to external environmental conditions by accumulating more P elements in nutrient-depleted soils. Compared to the three halophytes, *A. sinensis* is more adapted to a stable environment, while *S. salsa* is more advantageous in a variable environment.

## Data availability statement

The original contributions presented in the study are included in the article/supplementary material. Further inquiries can be directed to the corresponding author.

## Author contributions

YZ and TL analyzed the data and wrote the manuscript. JL performed the experiment, TL and JS designed the experiment. PZ participated in the sample plot survey. All authors contributed to the article and approved the submitted version.

## Funding

The funding was supported by the National Natural Science Foundation of China (41871089, 41971119, and 42171059), the “Collection, Conservation, and Accurate Identification of Forest Tree Germplasm Resources” of Shandong Provincial Agricultural Elite Varieties Project (2019LZGC01805), the Natural Science Foundation of Shandong Province (ZR2019MD024, ZR2020QD004), and the Science and Technology Support Plan for Youth Innovation of Colleges and Universities (2019KJD010).

## Conflict of interest

The authors declare that the research was conducted in the absence of any commercial or financial relationships that could be construed as a potential conflict of interest.

## Publisher’s note

All claims expressed in this article are solely those of the authors and do not necessarily represent those of their affiliated organizations, or those of the publisher, the editors and the reviewers. Any product that may be evaluated in this article, or claim that may be made by its manufacturer, is not guaranteed or endorsed by the publisher.
